# A Modular System to Evaluate the Efficacy of Protease Inhibitors against HIV-2

**DOI:** 10.1371/journal.pone.0113221

**Published:** 2014-11-24

**Authors:** Mohamed Mahdi, Krisztina Matúz, Ferenc Tóth, József Tőzsér

**Affiliations:** Laboratory of Retroviral Biochemistry, Department of Biochemistry and Molecular Biology, Faculty of Medicine, University of Debrecen, Debrecen, Hungary; NCI-Frederick, United States of America

## Abstract

The human immunodeficiency virus (HIV) protease is a homodimeric aspartyl protease that is crucial for the viral life-cycle, cleaving proviral polyproteins, hence creating mature protein components that are required for the formation of an infectious virus. With diagnostic measures and clinically used protease inhibitors focusing on HIV-1, due to its higher virulence and prevalence, studies of the efficacy of those inhibitors on HIV-2 protease remain widely lacking. Utilizing a wild-type HIV-2 vector backbone and cloning techniques we have developed a cassette system where the efficacy of clinically used protease inhibitors can be studied for various serotypes of HIV-2 protease both in enzymatic and cell culture assays. In our experiments, optimization of the expression protocol led to a relatively stable enzyme, for cell culture assays, the efficiency of transfection and transduction capability of the modified vector was tested and was not found to differ from that of the wild-type, moreover, a 2^nd^ generation protease inhibitor was used to demonstrate the usefulness of the system. The combination of assays performed with our cassette system is expected to provide an accurate measure of the efficacy of currently used; as well as experimental protease inhibitors on HIV-2.

## Background

The human immunodeficiency virus (HIV) is the causative agent of acquired immune deficiency syndrome (AIDS) that had claimed the lives of millions worldwide. Bearing a striking genomic resemblance to HIV-1, HIV-2 harbors a distinct clinical feature in that it is less infectious, less pathogenic and has a much lower rate of progression into AIDS [1]; moreover, HIV-2 has a much lower prevalence than its counter-part and has been localized mainly in a region confined to Western Africa. All those features have led to protease inhibitors (PIs) being designed specifically for HIV-1, with the majority of studies focusing on the efficacy of those inhibitors in association with HIV-1 protease, assuming that the inhibitors might have equal efficacy on both proteases [2], and that inadvertently had led to a scarcity in the field of studies of those inhibitors in association with HIV-2 protease. Molecular clock analysis studies have traced the origin of HIV-2 to the region of Guinea-Bissau as a result of an evolution from the simian immunodeficiency virus/sooty mangabeys [3]. So far eight groups of HIV-2 have been described in the literature and designated alphabetical letters A-H [4]–[6] with groups A and B gaining a wide international spread whereas the other subtypes remain relatively constrained in the West African region. The ending of the colonial era, population migration and international trade have all been implicated in the worldwide spread of HIV-2, especially in Europe, with countries such as France and Portugal reporting the incidence of HIV-2 being continuously on the rise [7], [8], while a slow decline in prevalence in West Africa being attributed to the low pathogenicity of HIV-2 has been observed [9]–[12].

Similar to that of HIV-1, HIV-2 protease is also a homodimeric aspartyl enzyme that is comprised of two identical monomers, each containing 99 amino acid residue, playing a vital role in the HIV life-cycle through processing of Gag and Gag-Pro-Pol precursor polyproteins leading to viral maturation. The literature describes a 39–48% similarity of amino acid sequences between HIV-1 and HIV-2 proteases [2], [13], depending on the virus subtype being studied. This polymorphism greatly affects the specificity of the protease for peptide substrates and inhibitors, hence leading to drug resistance [14]–[16], and may render an otherwise very potent inhibitor on HIV-1 protease obsolete. Despite the seemingly significant sequence differences between HIV-1 and -2 protease, the major structural elements around the active site remain highly conserved [13], and PI-associated mutations in HIV-2 having relatively high frequency are perhaps natural polymorphisms in their respective strains [17].

In comparison to HIV-1, fewer studies have examined the efficacy of clinically used protease inhibitors on HIV-2, moreover, researches have focused on either enzymatic [18] or *in vitro* cell culture studies on protease inhibitors in regards to HIV-2 [15], [19], [20], and significantly different IC50 and Ki results can be obtained in these assays. In kinetic assays, the results are highly influenced by the level of purity and degree of stability of the enzyme, results obtained from *in vitro* cell culture experiments on the other hand, are dependent on the nature of assay used and the types of cells under study. In addition, even a slight variation in the protease coding sequence may alter the results dramatically due to a variation of folding and stability of the enzyme; hence the use of different protease sequences may yield variable results. The lack of a standardized protocol to examine the efficacy of protease inhibitors remains as a hindrance in the analysis, therefore, development of a cassette system that facilitates the analysis of the potency of protease inhibitors on the same protease coding sequence both in kinetic and *in vitro* cell culture assays will greatly aid the determination of the PI's efficacy, and may help the design of inhibitors being more potent on HIV-2 protease.

## Materials and Methods

### HIV-2 vector system

The vector system is composed of HIV-2CGP as a structural protein expression construct, CRU5SINCGW a minimal HIV-2 vector with GFP expression cassette and pMD.G vector, coding for the envelope protein of vesicular stomatitis virus. HIV-2CGP and CRU5SINCGW were a kind gift from Joseph P. Dougherty at the Robert Wood Johnson Medical School [21].

### Cassette construction

HIV-2CGP protease coding sequence was used as template: 5'- CCT CAA TTC TCT CTT TGG AAA AGA CCA GTA GTC ACA GCA TAC ATT GAG GGT CAG CCA GTA GAA GTT TTG TTA GAC ACG GGA GCT GAC GAC TCA ATA GTA GCA GGA ATA GAG TTA GGA AAC AAT TAT AGC CCA AAA ATA GTA GGG GGA ATA GGG GGA TTC ATA AAT ACC AAG GAA TAT AAA AAT GTA GAA ATA GAA GTT CTA AAT AAA AAG GTA CGG GCC ACC ATA ATG ACA GGC GAC ACC CCA ATC AAC ATT TTT GGC AGA AAT ATT CTG ACA GCC TTA GGC ATG TCA TTA AAT CTA -3'. Site-directed mutagenesis was done according to QuikChange mutagenesis protocol (Stratagene, La Jolla, CA, USA), utilizing designed oligonucleotide primers: 5'-CTC TCT TTG GAA AAG ACC GGT AGT CAC AGC ATA C-3' and 5'-GGC AGA AAT ATT CTG ACA GCG CTA GGC ATG TCA TTA AAT CTA C-3' to introduce unique restriction sites AgeI and AfeI at 5′ and 3′ of the CGP protease coding region, respectively. The silent mutations were 8 amino acids apart from the ends of the protease coding sequence. NdeI and BamHI restriction sites were then attached to its 5′ and 3′ end respectively using PCR with the aid of designed oligonucleotides: 5'-CTT CAT ATG CCT CAA TTC TCT CTT TGG AAA AGA CCG G-3' and 5'-CGA TCC GTA CAG TAA TTT AGA TAC TCC TAG GGC G-3'. The entire region was then ligated into a pET11a expression plasmid (Invitrogen) for bacterial expression. Success of the mutagenesis and ligation were verified by restriction endonuclease enzyme digestion and DNA sequencing.

### 293T cell transfection and transduction

In accordance with an HIV-1 transfection protocol [22], 293T human embryonic kidney cells (Invitrogen) were seeded in T75 flask in 15 ml Dulbecco's Modified Eagle's Medium (DMEM) (Sigma-Aldrich) supplemented with 10% fetal bovine serum (FBS), 1% glutamine and 1% penicillin-streptomycin. The day before the transfection, cells were passaged in order to achieve 70% confluency the next day. At 70% confluency (5−6×10^6^ cells/ml) a total of 45 µg plasmid DNA was used for the transfection of cells using Polyethylenimine. Cells were then incubated at 37°C, 5% CO_2_ in 5 ml 1% FBS containing DMEM without antibiotics. After 6 hours, the medium was replaced by 15 ml DMEM containing 10% FBS, 1% glutamine, 1% penicillin-streptomycin. The medium was then collected after 24, 48 and 72 hours, filtered through a 0.45 µm polyvinylidene fluoride filter (Millipore) and concentrated by ultracentrifugation (100000 g, 2 hours, 4°C). The pellet containing viral particles was then dissolved in 200 µl phosphate-buffered saline (PBS) and stored at −70°C. ELISA based colorimetric reverse transcriptase assay (Roche) was then used to detect the amount of reverse transcriptase (RT) in the viral samples.

Regarding the infectivity (transduction) assay, 293T cells were plated in 96-wells plate in 200 µl DMEM supplemented with 10% FBS, 1% glutamine and 1% penicillin-streptomycin. At 50% confluency (2.5−3×10^4^ cells/ml), cells in 50 µl DMEM were infected with viral particles containing 57 ng reverse transcriptase as determined by the reverse transcriptase colorimetric assay per well. On the next day the medium was supplemented with 120 µl of DMEM containing 20% FBS, 2% glutamine, 2% penicillin-streptomycin and then the cells were incubated at 37°C, 5% CO_2_ for 6 days. Cells were then scraped off and fixed in PBS containing 1% formaldehyde. Infected cells were then checked under fluorescence microscope (Axiom 200) for the presence of green fluorescent protein (GFP). For quantitative analysis the cells were counted by flow cytometry (FACS Calibur, BD Bioscience) to determine the percentage of GFP positivity in 5000 cells.

### *In vitro* protease expression and purification

The protease ligated into pET11a was expressed in a culture of 100 ml of E. *coli* BL21(DE3) (Invitrogen) cells in Luria-Bertani medium supplemented with 100 µg/ml ampicillin. Culture was then induced at 0.8–1.0 OD_600nm_ with a final concentration of 1 mM isopropyl-B-D-1-thiogalactopyranoside for 3 hours. Cells were harvested by centrifugation at 5500 g (Beckman centrifuge, JA-14 rotor), 20 minutes, 4°C and the pellet was stored at 4°C overnight. The following day the pellet was dissolved in 8 ml buffer A (50 mM tris-hydroxymethyl aminomethane (Tris), 10 mM dithiothreitol (DTT), 10 mM ethylenediaminetetraacetic acid (EDTA), pH 8.0), after the addition of 4 mg of lysozyme the cells were disrupted using the sonication method (Branson Sonicator, 3×3 minutes at 40% energy, 4°C) and then centrifuged at 48000 g (JA-20 rotor) for 20 minutes at 4°C (step 1). The pellet was re-suspended in 8 ml buffer B (50 mM Tris, 10 mM DTT, 10 mM EDTA, 1 M urea, 0.5% Triton X-100, pH 8.0) followed by another centrifugation (step 2). On the following step the pellet was re-suspended in 8 ml buffer A and a repeat centrifugation was carried (step 3). The insoluble fraction was then solubilized in 5 ml buffer C (50 mM Tris, 10 mM DTT 5 mM EDTA, 7.5 M guanidine-HCl, pH 8.0) (step 4) [23]. The protease was then purified from this fraction using reversed-phase high performance liquid chromatography with the aid of an ÄKTA purifier (Amersham Pharmacia Biotech) using a POROS 20 R2 (PE Biosystems, PerSeptive Biosystems) C_18_ column. A linear gradient from 99.95% water and 0.05% trifluroacetic acid (TFA) to 40% acetonitril and 0.05% TFA over a period of 25 minutes was applied at a flow rate of 1 ml/min at room temperature. Following the elution, the peak fractions were immediately lyophilized with eppendorf concentrator plus and then solubilized in buffer D (20 mM piperazine-1, 4-bis(2-ethanesulfonic acid) (PIPES), 1 mM EDTA, 100 mM NaCl, 10% glycerol, 0.5% Nonident P40, pH 7.0) then pooled. The pooled fraction was dialyzed against the same buffer overnight and then concentrated using Amicon Ultra-4 centrifugal filter units (Millipore). Protein concentration of fractions was determined by the Bradford assay (Bio-Rad).

### Activity measurement

The activity of the protease was measured using an HPLC-based method utilizing an oligopeptide substrate MSLNLPVAKV as described previously [24]. The reaction contained 10 µl buffer E (0.5 M phosphate, 10 mM DTT, 4 M NaCl, 10% glycerol, pH 5.6), 5 µl substrate (2 mg/ml) dissolved in water and 5 µl purified protease. The reaction mixtures were incubated for 1 hour at 37°C, then the reaction was stopped by the addition of 180 µl 1% TFA and product and substrate peaks were separated by using a water-acetonitrile gradient in the presence of 0.05% TFA. Active site titration was performed as described [25] using darunavir as a potent inhibitor and the HPLC method.

### Inhibitory effect of darunavir

Following the verification of the enzyme activity, the second generation protease inhibitor darunavir (NIH AIDS Reagent Program) was used to test our modular system. Serial dilutions were prepared from the inhibitor using dimethyl sulfoxide (DMSO) in concentrations ranging from 10 nM to 50 µM, the catalytic reactions contained 10 µl buffer E, 4.8 µl substrate, 5 µl purified protease and 0.2 µl inhibitor, followed by incubation at 37°C for 1 hour. The concentration of the protease was adjusted to achieve less than 20% substrate hydrolysis. Reactions were terminated by the addition of 180 µl 1% TFA, thereafter HPLC measurements were used to determine the inhibitor's IC50 by measuring the decrease in substrate hydrolysis. The inhibitory constant K_i_ was then calculated from IC50 using the formula K_i_ =  (IC50- E/2)/(S/K_m_+1), in which E is the active enzyme concentration, S is the substrate concentration and Km is the Michaelis constant.

In the cell culture experiments, 293T cells in T75 flask were transfected using the protocol mentioned above. After 5-6 hours incubation at 37°C, cells were split and transferred into a 96-wells plate containing serial dilutions of darunavir ranging from 0.05–100 µM in a total volume of 200 µl DMEM/well supplemented with 10% FBS, 1% glutamine and 1% penicillin-streptomycin. After 3 days incubation at 37°C, the virus containing medium was collected from the wells, briefly centrifuged to remove cellular debris, and 10 µl samples were taken from each corresponding well. Reverse transcriptase colorimetric assay was then used to calculate the IC50 from triplicate measurements. It is necessary to mention that in order to get accurate results using the colorimetric assay, a slight modification to the manufacturer's protocol was needed, such as the incubation of samples with reaction mixture for 17–18 hours, to allow for sufficient detection and quantification of reverse transcriptase.

### Study of autodegradation/autoinactivation

To examine the stability of the purified protease and its susceptibility to autodegradation, the active protease dialyzed at 4°C against buffer D (pH 7.0) was incubated at 37°C for various time intervals and the remaining activity was measured as described for the activity assays. Similar experiments were carried out with purified protease dialyzed in 50 mM Na-acetate and 50 mM NaCl, (pH 5.0) [23]. For SDS-polyacrylamide gel analysis, 15 µl of protease was incubated at 37°C for multiple time intervals, then run on 16% SDS gel, densitometry was then used to determine the density of the different protease bands using AlphaImager HP system software.

## Results and Discussion

**Figure 1:**
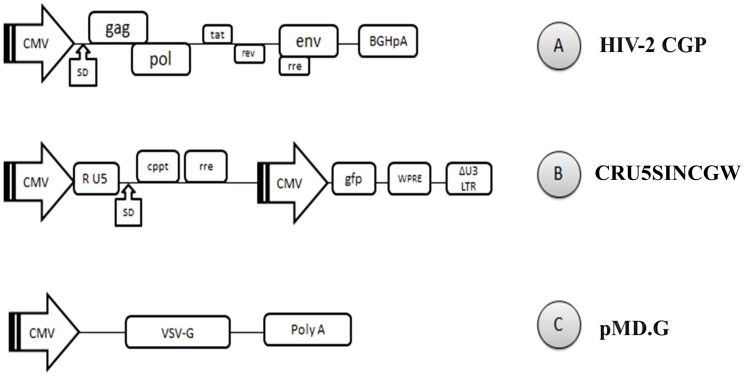
Vectors used for 293T cell transfection. **A**: HIV-2CGP, a CMV driven gag-pol expression construct. **B**: CRU5SINCGW, HIV-2 based minimal vector with a GFP expression cassette. **C**: pMD.G a plasmid coding for VSV envelope protein. CMV: human cytomegalovirus immediate early promoter; SD: splice donor site; rre: rev response element. BGHpA: bovine growth hormone polyadenylation signal. U3-R-U5; retroviral long terminal repeats. cppt; central polypurine tract. GFP; green fluorescence protein. WPRE; woodchuck hepatitis virus post-transcriptional regulatory element. VSV-G; vesicular stomatitis virus G protein. PolyA; polyadenylation site.

**Figure 2:**
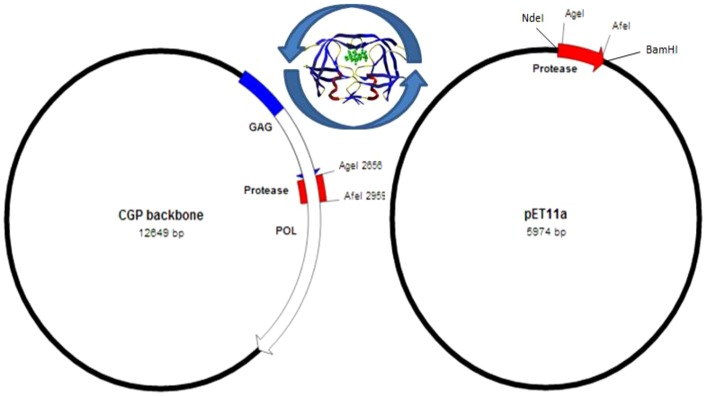
Schematic representation of the cassette system. HIV-2 protease can be interchanged between the lentiviral HIV-2CGP vector and the *in vitro* pET11a expression plasmid, utilizing the unique restriction sites. Protease sequence: P Q F S L W K R * P V V T A Y I E G Q P V E V L L D T G A D D S I V A G I E L G N N Y S P K I V G G I G G F I N T K E Y K N V E I E V L N K K V R A T I M T G D T P I N I F G R N I L T * A L G M S L N L. The asterisks indicate the beginning and end of the cassette.

**Figure 3:**
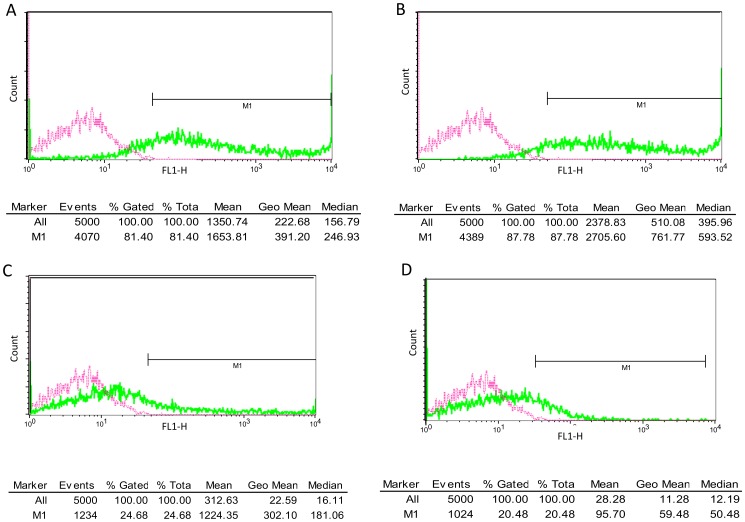
Flow cytometric detection of green fluorescent protein. **A**: number of gated cells following transfection using wild-type HIV-2 vector. **B**: number of gated cells following transfection with the vector modified for the cassette system. **C**: transduction using wild-type vector. **D**: transduction using the modified vector. A minimum of three experiments were carried and the data shown are representative samples of results acquired. Pink color indicates control cells.

In this paper, we present an HIV-2 cassette system that renders the study of the HIV-2 protease possible both in *in vitro* kinetic and cell culture studies for comparative analysis. Utilizing a ROD strain based HIV-2 lentiviral vector system (Figure 1), unique silent restriction sites were introduced into the protease coding region 8 amino acids apart from the termini that allows for the interchange of different protease coding segments (Figure 2). Analysis of HIV-2 protease sequences have shown that the majority of strains harboring treatment-associated resistance mutations comprise a single or multiple amino acid changes that fall within that region [17], [26], therefore, the positioning of the silent restriction sites will allow for the extensive study of those mutations and their role in the susceptibility to PIs. Having optimized the transfection and transduction protocols in the cell culture experiments, we have achieved more than 87% transfection and 25% transduction efficiency as measured by flow cytometry detecting GFP positive cells (Figure 3).

Calculation of viral titers by multiplying the cell number, the percentage of GFP and the dilution factor from transduction experiments yielded infectious unit/ml (IU/ml) of 2.2×10^6^ for the wild-type, and 1.83×10^6^ for the modified vector, those results fall within the expected transduction efficiency of HIV-2 derived SIN vectors in adherent cell lines [21], [27]. It is also noteworthy that the percentage of positive GFP cells was related directly to the concentration of recombinant virus used. In our experiments, transduction was merely used as a measure to compare the modified vector to the wild-type, the 20–25% efficiency was calculated as the percentage of GFP positive cells detected by flow cytometry, as a result of using the same concentration of viruses to transduce 293T cells in a 96-wells plate.

The vector used is an HIV-2 based self-inactivating vector, due to alterations of the LTR regions. As a result, there is a loss of transcriptional activity following genome integration into target cells, rendering the virus capable of only a single round of replication, therefore, due to the fact that the viral protease is essential to the processing of the viral polyproteins in the late stage of infection, inhibition profiling of protease inhibitors was performed in the virus production stage, using reverse transcriptase as a measurement to detect the efficacy of the PI. To support our methodology, previous studies had shown that the protease is required for the full activity of the reverse transcriptase [28], thus, using protease inhibitors at this stage is expected to result in the formation of immature, non-infectious virus particles, with decreased RT activity; depending on the concentration of PI used. Levels of RT can then be detected by the reverse transcriptase colorometric assay, and provide a valid IC50 values for various PIs.

In single cycle phenotypic assays, commonly, either luciferase activity or GFP fluorescence can be measured after infection of target cells, alternatively, RT activity can be quantified from the supernatant, as a measure of mature, infectious particles [15], [21], [29]. We chose to detect RT since it is a significantly more sensitive measure as compared to GFP fluorescence.

**Figure 4:**
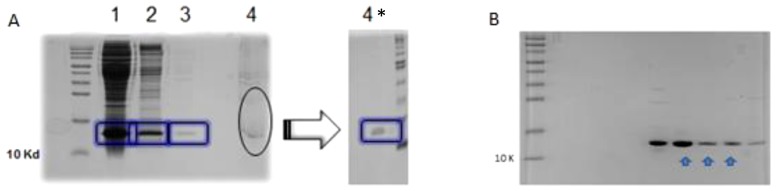
SDS-polyacrylamide gel electrophoresis (16%). **A**: supernatants 1, 2, 3, 4 obtained from steps 1-4 following expression in BL21(DE3) cells. 15 µl of supernatant was run on 16% SDS-polyacrylamide gel, the asterisk indicates the solubilized pellet after 15x dilution with water to assist visualization. **B**: protease fractions after reversed-phase high performance liquid chromatography, arrows indicate the pooled fractions.

**Figure 5:**
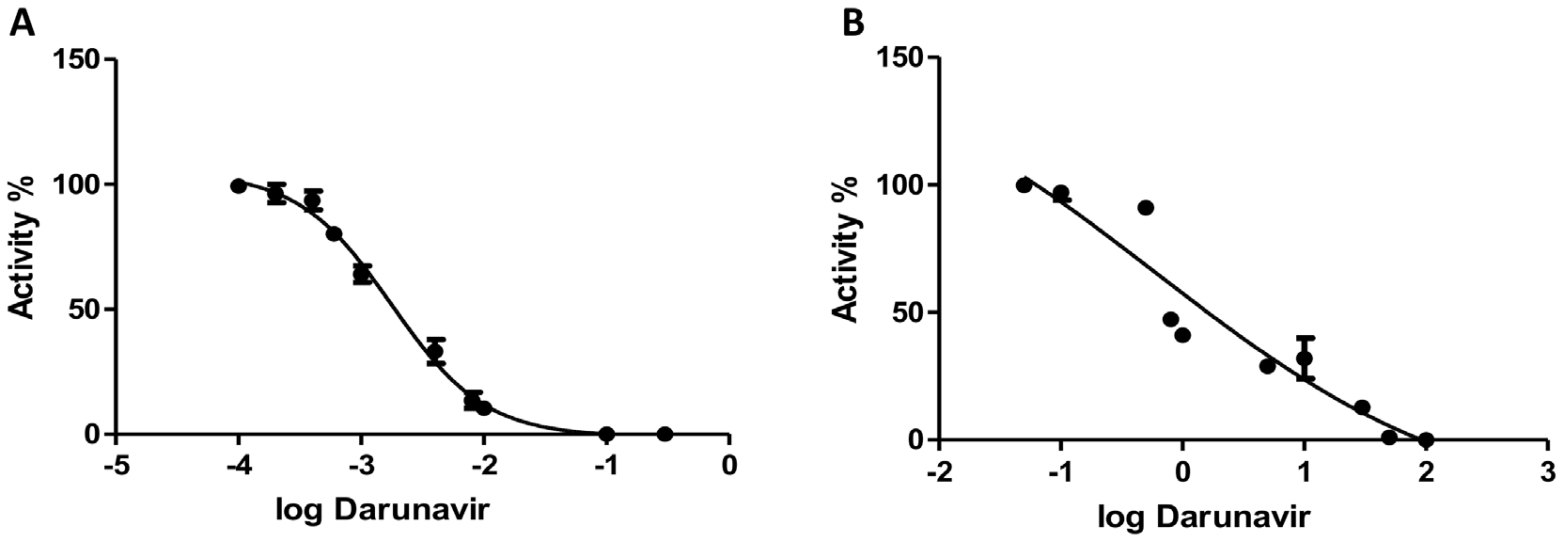
Inhibitory effect of darunavir. **A**: IC_50_ determination *in vitro* using HPLC. **B**: IC_50_ determination in cell culture. Activity percentage is plotted on the Y axis *versus* logarithmic transformation of the inhibitor's concentration (µM).

To provide a purified enzyme for the kinetic analysis, the protease from the CGP plasmid was amplified and ligated into the expression vector pET11a. After expression in E. *coli*, the protease was purified using HPLC with the aid of a C18 column (Figure 4). To determine the activity of the purified protease, it was incubated with an oligopeptide substrate MSLNL↓PVAKV representing the protease/reverse transcriptase cleavage site in HIV-2 (arrow marks the site of cleavage) and HPLC was used to analyze the cleavage products as described previously [24]. Following the confirmation of the activity of the enzyme, measurements were then performed to determine the kinetic parameters. The obtained values K_m_ = 0.012 (±0.002) mM, k_cat_ = 0.91 (±0.02) s^−1^, k_cat_/K_m_ = 75.8 (±12.7) mM^−1^s^−1^ were very similar to those previously determined for a chemically synthesized, wild-type enzyme, refolded using the same protocol [24]. Based on the protein content and activity of the protease samples, the folding efficiency was approximated to be 10–15%. In order to demonstrate the usefulness of the modular system, a second generation PI (darunavir) was used for kinetic and cell culture inhibition, HPLC measurements performed in triplicates were used to determine the IC50 which was found to be 0.002 (±0.0001) µM that is equivalent to Ki = 0.05 (±0.005) nM values, while experiments done *in vitro* using cultured cells yielded an IC50 of 0.42 (±0.05) µM (Figure 5). These results are in the same range with those found in previous studies [18], other experiments conducted solely on cell culture yielded slightly different values that maybe a result of using different culture cells or the type of assay used [19].

Autoproteolysis of the HIV proteases is best described as a process by which the protease domain facilitates a cascade of proteolytic reactions that ultimately lead to the dissociation of the free mature protease that in turn cleaves different peptide sequences in the Gag and Gag-Pol polyproteins [30]. Following its dissociation, this autolytic ability of the viral protease also plays a major role in its autodegradation. It is thought that this degradation occurs initially at an exposed amino-terminal strand/loop that is likely to be exposed to the protease in case of HIV-1/2 and SIV [31]. Autodegradation is a major factor in the attributed loss of activity of the viral enzyme, a process that had proven to be a major hindrance in the expression and purification of the enzyme. Methods aimed at decreasing the autodegradation have been used such as the use of catalytic-site inhibitors, storing the protease at a suboptimal pH, or modifying the implicated amino acid cleavage sites (Leu5-Trp6 in case of HIV-1), which has been found to be more resistant to autodegradation in HIV-2 [31].

**Figure 6:**
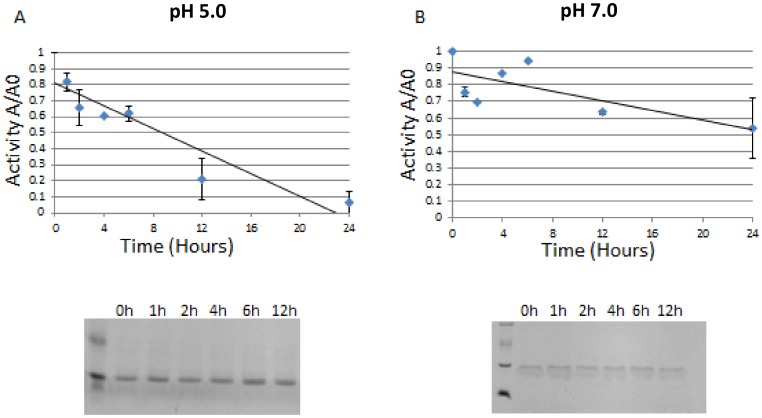
Studies on autodegradation of HIV-2 protease. **A**: autodegradation analysis in a buffer composed of 50 mM sodium acetate and 50 mM sodium chloride (pH 5.0). **B**: autodegradation in buffer D (pH 7.0). X axis represents the incubation time in hours, Y axis represents A/A0 the ratio of active protein A at a given time to the total active protein A0 at time  = 0. Autodegradation characterization was also performed on 16% SDS gel electrophoresis, the corresponding gel pictures are shown, the seemingly double banding seen in SDS-gel picture of B is due to artifact caused by the PR buffer composition.

In our activity analysis of the protease, we have found that incubation in a buffer having a neutral pH greatly decreases its autodegradation/autoinactivation, as evident from our comparative analysis with incubation in an acidic (pH 5.0) buffer that was typically used previously to dialyze HIV proteases. When the activity of the enzyme was monitored, the protease in the buffer having neutral pH maintained almost half of its activity after a 24 hour incubation period at 37 °C, in contrast to incubation at pH 5.0, which yielded minimal activity after only 12 hours of incubation (Figure 6). As the SDS-gel analysis did not show substantial protein degradation following either refolding protocol, the loss of enzymatic activity appears to be majorly the consequence of autoinactivation rather than autodegradation. We also found that the immediate lyophilization and storage in a pH 7.0 buffer after reversed-phase chromatography aided greatly in the preservation of its activity, and facilitated the prolonged storage and studies on the purified fractions (data not shown).

The success of this modular system in testing the efficacy of darunavir both in kinetic and in cell culture assays has only paved the way for future measurements of currently widely used protease inhibitors, given the absence of a standardized protocol and the antigenic variability of clinical isolates. We hope that the development of this cassette system and the optimization of the protease expression may aid in the thorough analysis of HIV-2 protease and its susceptibility to protease inhibitors in clinical use.
